# A ZIP-Active Tumor Cell State Defines an NRF2-Associated Ferroptosis-Resistant and Immune-Cold Transcriptional Program in Head and Neck Squamous Cell Carcinoma

**DOI:** 10.3390/cancers18193104

**Published:** 2026-09-24

**Authors:** Hideyuki Takahashi, Miho Uchida, Kaoru Takahashi, Hiroyuki Hagiwara, Hiroe Tada, Toshiyuki Matsuyama, Kazuaki Chikamatsu

**Affiliations:** Department of Otolaryngology-Head and Neck Surgery, Gunma University Graduate School of Medicine, 3-39-22 Showa-machi, Maebashi 371-8511, Gunma, Japantikamatu@gunma-u.ac.jp (K.C.)

**Keywords:** head and neck squamous cell carcinoma, zinc transporter, single-cell RNA sequencing, NRF2, ferroptosis, oxidative stress, tumor microenvironment, transcriptomics

## Abstract

**Background/Objectives**: Zinc homeostasis has emerged as an important regulator of cancer biology, yet the biological and clinical significance of zinc transporter-associated transcriptional programs in head and neck squamous cell carcinoma (HNSCC) remains poorly understood. This study aimed to identify zinc transporter-associated epithelial tumor cell states and evaluate their molecular and clinical significance. **Methods**: Single-cell RNA sequencing data from an HNSCC cohort were analyzed to identify epithelial tumor cell populations associated with zinc transporter expression and to characterize their molecular features. A ZIP-active transcriptional signature was subsequently applied to The Cancer Genome Atlas (TCGA)-HNSCC cohort to evaluate biological characteristics, immune features, survival outcomes, and the influence of *NFE2L2*/*KEAP1/CUL3* mutation status. **Results**: A distinct epithelial tumor cell population characterized by coordinated upregulation of *SLC39A3* and *SLC39A4* was identified and designated the ZIP-active tumor cell state. This population showed NRF2-associated oxidative stress adaptation, glutathione metabolism, and ferroptosis suppression, together with increased NRF2 activity. In the TCGA-HNSCC cohort, a high ZIP-active score was associated with oxidative stress adaptation, reduced T-cell-related immune signatures, and poorer overall survival. *NFE2L2/KEAP1/CUL3*-mutant tumors showed higher ZIP-active and NRF2-related scores than wild-type tumors, while NRF2-related metabolic features remained associated with ZIP activity in both groups. **Conclusions**: The ZIP-active tumor cell state represents an NRF2-linked metabolic program associated with oxidative stress adaptation, immune alterations, and adverse prognosis in HNSCC. Its persistence across *NFE2L2/KEAP1/CUL3* mutation groups suggests that ZIP activity reflects NRF2-related tumor biology beyond genomic alterations alone.

## Simple Summary

Zinc is an essential nutrient that helps regulate many cellular functions, but its role in head and neck cancer remains incompletely understood. By analyzing single-cell and large-scale patient gene-expression datasets, we identified a previously unrecognized group of tumor cells characterized by coordinated expression of the zinc transporters *SLC39A3* and *SLC39A4*, termed the ZIP-active tumor cell state. These cells showed molecular features associated with adaptation to oxidative stress, altered antitumor immune features, and poor patient survival. Tumors with *NFE2L2/KEAP1/CUL3* mutations showed higher ZIP-active and NRF2-related activity; however, NRF2-related metabolic features remained associated with ZIP activity in both wild-type and mutant tumors. Our findings provide new insight into how zinc-related pathways contribute to tumor progression and suggest that the ZIP-active state reflects NRF2-related tumor biology extending beyond genomic alterations alone.

## 1. Introduction

Head and neck squamous cell carcinoma (HNSCC) is the sixth most common malignancy worldwide and remains associated with substantial morbidity and mortality despite advances in surgery, radiotherapy, systemic therapy, and immunotherapy [1,2,3]. Clinical outcomes vary considerably among patients with apparently similar clinicopathological characteristics, reflecting the marked biological heterogeneity of HNSCC [2]. Recent advances in single-cell RNA sequencing (scRNA-seq) have revealed diverse epithelial tumor cell states associated with proliferation, differentiation, metabolism, and treatment response, highlighting intratumoral heterogeneity as a major determinant of tumor behavior [2,4,5]. However, the transcriptional programs underlying metabolic adaptation and stress resistance within malignant epithelial cells remain incompletely understood.

Zinc is an essential trace element that participates in numerous biological processes, including cell proliferation, antioxidant defense, DNA repair, and regulation of transcriptional activity [6,7,8,9]. Intracellular zinc homeostasis is tightly regulated by two complementary transporter families: the ZIP (SLC39) family, which increases cytosolic zinc availability, and the ZnT (SLC30) family, which reduces cytosolic zinc through efflux or intracellular sequestration [10,11]. Dysregulated expression of zinc transporters has been implicated in tumor initiation and progression across multiple cancer types, and several members of the ZIP family, particularly *SLC39A4* (ZIP4), have been associated with enhanced proliferation, invasion, and poor clinical outcomes [10,12,13]. Nevertheless, most previous studies have focused on individual zinc transporters, whereas it remains unclear whether coordinated zinc transporter activity defines biologically distinct tumor cell states within HNSCC.

Among the transcriptional programs that enable tumor adaptation, the NRF2 pathway is a master regulator of oxidative stress responses and metabolic reprogramming [14,15,16]. Persistent NRF2 activation promotes antioxidant defense, glutathione metabolism, and resistance to oxidative stress and has been associated with poor prognosis and therapeutic resistance in multiple malignancies, including HNSCC [15,17,18,19,20]. NRF2 also contributes to transcriptional programs associated with ferroptosis suppression and has recently been implicated in shaping the tumor immune microenvironment [18,21,22,23]. Although activating alterations in *NFE2L2, KEAP1*, or *CUL3* represent well-established mechanisms of NRF2 activation, accumulating evidence suggests that NRF2-associated transcriptional programs may also arise through non-genetic mechanisms [15,17,24]. Whether zinc transporter-associated transcriptional programs are linked to NRF2 activation, ferroptosis-related biology, and clinical outcomes in HNSCC has not been systematically investigated.

In our previous single-cell analysis of the HNSCC cohort, we observed preferential expression of *SLC39A2*, *SLC39A3*, and *SLC39A4* within epithelial tumor cells, particularly in proliferating epithelial populations [25]. Building on these observations, we aimed to determine whether coordinated ZIP transporter expression defines a distinct epithelial tumor cell state in HNSCC and to evaluate its biological characteristics, clinical relevance, and relationship with canonical *NFE2L2*/*KEAP1/CUL3* mutations through integrated analyses of single-cell and bulk transcriptomic datasets.

## 2. Materials and Methods

### 2.1. Single-Cell RNA Sequencing Dataset

Single-cell RNA sequencing data were retrieved from the Gene Expression Omnibus under accession number GSE164690. The dataset includes CD45-positive and CD45-negative cells isolated from freshly resected primary head and neck squamous cell carcinoma specimens from 14 tumors, including human papillomavirus (HPV)-positive and -negative cases. Clinical characteristics of this cohort have been reported previously in the original study and in our previous study [4,25]. All single-cell analyses were conducted in R (version 4.3.1) using Seurat version 5. Available clinical characteristics of the patients included in the single-cell RNA-sequencing cohort are summarized in Supplementary Table S1.

### 2.2. Preprocessing and Purification of Epithelial Cells

Cells expressing fewer than 100 genes were excluded prior to analysis. Gene-expression counts were normalized using Seurat’s global-scaling normalization method with a scale factor of 10,000, and the 2000 most variable genes were selected for downstream analyses. Graph-based clustering was performed following dimensionality reduction, and cellular distributions were visualized using Uniform Manifold Approximation and Projection (UMAP). Cluster marker genes were identified using the FindAllMarkers function. To facilitate cell-type annotation, normalized expression values were averaged across cells within each cluster to generate cluster-level expression profiles, which were subsequently analyzed using the xCell R package (version 1.1.0). Cluster annotations were further confirmed by examining canonical lineage-marker genes.

Epithelial cells were extracted from the integrated Seurat object and independently re-clustered. To minimize contamination by non-epithelial cells, cells expressing immune, fibroblast, or endothelial lineage markers or exhibiting high lineage-specific module scores were excluded, generating a purified epithelial-cell subset for downstream analyses. Patient-level pseudo-bulk expression profiles were generated by averaging log-normalized expression values across purified epithelial tumor cells from each patient. Spearman’s rank correlation analysis was performed to evaluate associations between zinc transporter expression and representative cancer-related transcriptional signatures (Supplementary Table S2). Cluster-specific expression patterns of zinc transporter genes were examined across epithelial subclusters. The epithelial cluster exhibiting the highest expression of *SLC39A3* and *SLC39A4* was designated the ZIP-active tumor cell state, and all remaining epithelial clusters were collectively defined as the reference population. Differentially expressed genes between ZIP-active and reference epithelial tumor cells were identified using Seurat’s FindMarkers function with a logistic regression model while adjusting for patient identity as a latent variable. Genes with an adjusted *p*-value < 0.05 and an average log2 fold change (avg_log2FC) ≥ 2.0 were retained to define the ZIP-active transcriptional signature. Genes were ranked according to the signed logarithm of the adjusted *p*-value and subjected to gene set enrichment analysis (GSEA) using the fgsea package (version 1.30.0) with Hallmark gene sets obtained from the Molecular Signatures Database (MSigDB). Transcription factor activity was inferred using the DoRothEA (version 1.16.0) regulon resource together with the VIPER (version 1.38.0) algorithm. Representative biological pathway scores were calculated at the single-cell level using predefined Hallmark, Reactome, and manually curated gene sets (Supplementary Table S3). Cellular differentiation trajectories were reconstructed using Slingshot (version 2.12.0). Cluster 6 was specified as the root population based on its transcriptional characteristics, and pseudotime values and lineage assignments were estimated to evaluate the emergence of the ZIP-active tumor cell state during epithelial tumor cell differentiation.

### 2.3. TCGA Validation Cohort

To validate the biological and clinical relevance of the ZIP-active transcriptional program identified by single-cell RNA sequencing, transcriptomic and clinical data from The Cancer Genome Atlas (TCGA) HNSCC cohort were analyzed. Illumina HiSeq RNA-seq V2 normalized gene-expression profiles and corresponding clinicopathological data for 520 patients were retrieved through FireBrowse (http://firebrowse.org/), including 97 HPV-positive and 423 HPV-negative tumors.

A ZIP-active score was generated from the differentially expressed genes defining the ZIP-active epithelial tumor cell state identified in the single-cell analysis. Genes meeting the thresholds of adjusted *p* < 0.05 and avg_log2FC ≥ 2.0 were used to define the ZIP-active transcriptional signature. Gene-expression values were standardized, and the ZIP-active score was calculated for each tumor using this gene signature. Correlations between the ZIP-active score and representative cancer-related transcriptional signatures were evaluated using Spearman’s rank correlation coefficient (Supplementary Table S2). Correlation analyses were restricted to variables with sufficient sample numbers, and *p*-values were adjusted using the Benjamini–Hochberg method.

For survival analyses, patients were stratified into ZIP-active-high and ZIP-active-low groups using endpoint-specific optimal receiver operating characteristic (ROC)-derived cutoffs. Kaplan–Meier survival curves were generated for overall survival and progression-free survival, and differences between groups were evaluated using the log-rank test. Multivariable Cox regression models were constructed to evaluate the independent prognostic significance of the ZIP-active classification after adjustment for selected clinical variables. Variables included in the multivariable models were selected according to their discriminative performance and statistical significance. Associations between clinicopathological characteristics and the ZIP-active score were also examined.

To characterize biological differences between ZIP-active-high and ZIP-active-low tumors, genes were ranked using the signal-to-noise metric and subjected to GSEA with the fgsea package using Hallmark gene sets obtained from MSigDB. Representative T-cell-related immune signatures were additionally compared between ZIP-active-high and ZIP-active-low tumors (Supplementary Table S4). Immune-cell fractions estimated by CIBERSORTx were also compared between ZIP-active-high and ZIP-active-low tumors.

The influence of canonical NRF2-pathway mutations was further evaluated by stratifying tumors according to the mutation status of *NFE2L2*, *KEAP1*, and *CUL3*. Signature correlations, survival analyses, pathway enrichment analyses, immune-signature comparisons, and immune-cell fractions estimated by CIBERSORTx were subsequently performed separately within the wild-type and mutant subgroups using the same analytical framework.

### 2.4. Statistical Analysis

Statistical analyses were performed using R (version 4.3.1). Depending on the specific analysis, comparisons of continuous variables between two groups were performed using Welch’s *t*-test or the Wilcoxon rank-sum test, while comparisons across three or more groups were performed using one-way analysis of variance (ANOVA). Correlations between continuous variables were evaluated using Spearman’s rank correlation coefficient. All tests were two-sided. Unless otherwise specified, *p*-values were adjusted for multiple comparisons using the Benjamini–Hochberg method, and an adjusted *p*-value < 0.05 was considered statistically significant.

## 3. Results

### 3.1. Identification of the ZIP-Active Epithelial Tumor Cell State

Building on our previous observation that *SLC39A2*, *SLC39A3*, and *SLC39A4* were preferentially expressed in epithelial tumor cells, particularly cycling epithelial cells, in the GSE164690 cohort, we first investigated whether the expression of these ZIP transporters was associated with representative tumor-related transcriptional programs. At the patient level, pseudo-bulk correlation analysis demonstrated that *SLC39A3* and *SLC39A4* showed broad positive correlations with multiple cancer-associated signatures, including proliferation, cell-cycle progression, reactive oxygen species (ROS) response, DNA repair, oxidative phosphorylation (OXPHOS), and glycolysis, whereas *SLC39A2* exhibited more limited associations (Figure 1A). These findings suggested that expression of specific ZIP transporters, particularly *SLC39A3* and *SLC39A4*, is linked to metabolically active and oxidative stress-adapted tumor cell phenotypes.

To determine whether these transcriptional characteristics were confined to a discrete epithelial tumor cell population, purified epithelial tumor cells were re-clustered and visualized by UMAP, resulting in 12 epithelial subclusters (Figure 1B). Among these, cluster 7 exhibited the highest combined expression of *SLC39A3* and *SLC39A4* and was therefore designated the ZIP-active tumor cell state (Figure 1C).

Comparison of ZIP transporter expression between the ZIP-active tumor cell state and the remaining epithelial tumor cells demonstrated significantly higher expression of *SLC39A4* and *SLC39A3* in the ZIP-active population, whereas *SLC39A2* expression did not differ significantly between the two groups (Figure 1D). These findings indicate that the ZIP-active tumor cell state is primarily characterized by coordinated upregulation of *SLC39A3* and *SLC39A4*, providing the basis for subsequent molecular and clinical analyses.

### 3.2. Molecular Characterization of the ZIP-Active Tumor Cell State

To characterize the molecular features of the ZIP-active tumor cell state, we first compared its transcriptional profile with that of the remaining epithelial tumor cells. Differential expression analysis revealed a distinct transcriptional profile in ZIP-active cells, with preferential expression of genes associated with cellular stress responses and redox regulation (Figure 2A). We therefore examined whether these transcriptional differences were accompanied by coordinated pathway-level alterations.

GSEA demonstrated that ZIP-active tumor cells were enriched for oxidative phosphorylation, reactive oxygen species pathway, MYC targets V1, apical surface, and NOTCH signaling, whereas TNFα signaling via NF-κB, IL6–JAK–STAT3 signaling, TGF-β signaling, KRAS signaling up, and coagulation were preferentially enriched in the remaining epithelial tumor cells (Figure 2B). These findings indicated that the ZIP-active state is characterized by a metabolically active and oxidative stress-associated transcriptional phenotype, accompanied by reduced enrichment of several inflammatory signaling programs.

We next sought to identify transcriptional regulators associated with this phenotype. DoRothEA/VIPER analysis identified NFE2L2 (NRF2) as the most strongly activated transcription factor in ZIP-active tumor cells, with additional activation of transcription factors including FOSL1, MAFG, BACH1, E2F family members, and MYC (Figure 2C). In contrast, several inflammatory transcriptional regulators, including RELA and NFKB1, showed greater inferred activity in the other epithelial tumor cells. These results pointed to NRF2-centered transcriptional regulation as a prominent feature of the ZIP-active state.

To further validate this association, we quantified predefined biological pathway scores across epithelial clusters. Cluster 7, corresponding to the ZIP-active state, showed prominent enrichment of ROS response, NRF2-like, KEAP1–NFE2L2, ferroptosis-suppressive, glutathione metabolism, and oxidative phosphorylation signatures (Figure 2D), and the NRF2-like score was spatially concentrated within the ZIP-active population on UMAP (Figure 2E). These findings were further supported by direct comparison of pathway scores between ZIP-active and other epithelial tumor cells, confirming enrichment of NRF2-associated oxidative stress and metabolic programs in the ZIP-active population (Supplementary Figure S1A). Collectively, these complementary analyses linked the ZIP-active phenotype to an NRF2-associated redox-adaptive program accompanied by enhanced glutathione metabolism and a transcriptional profile consistent with reduced ferroptosis susceptibility.

Finally, we investigated how this phenotype was positioned along epithelial tumor cell trajectories. Slingshot analysis identified a differentiation branch directed toward the ZIP-active cluster (Figure 2F), within a broader trajectory structure comprising seven inferred branches (Supplementary Figure S1B). Along the ZIP-active-directed branch, *SLC39A4* expression and ROS, NRF2-like, and OXPHOS pathway scores increased with pseudotime, whereas G2M activity showed a more transient pattern (Figure 2G). Branch-level comparison further demonstrated that the trajectory toward cluster 7 was distinguished by increased ZIP-transporter expression together with NRF2-associated genes and oxidative stress-related pathway activity relative to other epithelial lineages (Supplementary Figure S1C). These findings suggest that the ZIP-active phenotype is associated with progressive acquisition of an NRF2-linked oxidative stress-adaptive state along a distinct epithelial tumor cell trajectory.

### 3.3. Validation of the ZIP-Active Transcriptional Program in the TCGA Cohort

To determine whether the molecular characteristics of the ZIP-active tumor cell state identified by single-cell RNA sequencing were preserved at the patient level, we applied the ZIP-active transcriptional signature to the TCGA-HNSCC cohort. The resulting ZIP-active score showed positive correlations with NRF2-related signatures, glutathione metabolism, oxidative stress response, and ferroptosis-suppressive programs (Figure 3A,B, Supplementary Figure S2A), demonstrating that the transcriptional features identified in the ZIP-active epithelial tumor cell state were reproducible in bulk tumor transcriptomes. These findings indicate that the ZIP-active program represents a coordinated biological state characterized by enhanced oxidative stress adaptation rather than isolated upregulation of individual zinc transporter genes.

Next, patients were stratified into ZIP-active-high and ZIP-active-low groups using endpoint-specific optimal cutoffs. Kaplan–Meier analysis demonstrated that patients with ZIP-active-high tumors had significantly poorer overall survival than those with ZIP-active-low tumors (Figure 3C). Multivariable Cox proportional hazards analysis further confirmed that the ZIP-active classification remained independently associated with poorer overall survival after adjustment for established clinicopathological variables (Table 1). The ZIP-active score differed significantly according to primary tumor site and was higher in patients with advanced TNM stage, whereas no significant associations were observed with HPV status or individual T, N, and M factors (Supplementary Table S5). These findings suggest that the ZIP-active transcriptional program has prognostic significance for overall survival beyond conventional clinical factors.

To further characterize the global transcriptional landscape associated with the ZIP-active program, GSEA was performed using the ranked transcriptome of the TCGA cohort. ZIP-active-high tumors were enriched for Hallmark pathways related to reactive oxygen species, oxidative phosphorylation, xenobiotic and fatty acid metabolism, MYC targets, peroxisome, and mTORC1 signaling, whereas immune- and inflammation-related pathways, including interferon responses, inflammatory response, IL6–JAK–STAT3 signaling, TNFα signaling via NF-κB, complement, and allograft rejection, were preferentially enriched in ZIP-active-low tumors (Figure 3D). These findings indicate that the ZIP-active program is associated with enhanced metabolic activity and cellular stress adaptation accompanied by relative suppression of inflammatory signaling.

Because oxidative stress adaptation has been implicated in modulation of the tumor immune microenvironment, we next examined T-cell-related immune signatures. ZIP-active-high tumors consistently exhibited significantly lower T-cell-associated immune scores than ZIP-active-low tumors (Figure 3E,F), indicating that activation of the ZIP-active program is accompanied by an immune-cold tumor microenvironment. CIBERSORTx analysis further demonstrated differences in immune-cell composition between the two groups, including reduced CD8 T-cell and CD4 memory T-cell fractions in ZIP-active-high tumors (Figure 3G, Supplementary Figure S2B). Together, these findings demonstrate that the ZIP-active transcriptional program identified at single-cell resolution is associated with coordinated activation of NRF2-related oxidative stress adaptation, suppression of antitumor immunity, and poorer overall survival in HNSCC.

### 3.4. NFE2L2/KEAP1/CUL3 Mutation Status Modifies the Biological and Clinical Features Associated with the ZIP-Active Program

Because constitutive NRF2 activation in HNSCC is frequently associated with *NFE2L2*, *KEAP1*, and *CUL3* alterations, we next investigated whether the biological and clinical significance of the ZIP-active program depended on the mutational status of these canonical NRF2-pathway genes. Tumors harboring *NFE2L2*/*KEAP1/CUL3* alterations exhibited significantly higher ZIP-active scores together with increased NRF2-related, glutathione metabolism, ferroptosis-suppressive, ROS response, and OXPHOS signatures than wild-type tumors, whereas EGFR signaling was significantly reduced (Figure 4A). Additional comparisons of representative cancer-related signatures demonstrated that these differences were largely confined to redox- and metabolism-related programs, while many other tumor-associated transcriptional signatures showed no significant differences between the two groups (Supplementary Figure S3A). These findings indicate that canonical NRF2-pathway alterations are associated with enhancement of the ZIP-active/NRF2-related transcriptional phenotype rather than broad changes across tumor-associated transcriptional programs.

We next examined whether the relationship between the ZIP-active program and downstream biological features differed according to *NFE2L2*/*KEAP1/CUL3* mutation status. Stratified correlation analyses demonstrated that the ZIP-active score remained positively associated with NRF2-related, glutathione metabolism, ferroptosis-suppressive, ROS response, and OXPHOS signatures in both wild-type and mutant tumors, although the strength of individual associations varied between the two subgroups (Figure 4B). These findings indicate that the relationship between ZIP activity and NRF2-related metabolic programs is not restricted to tumors harboring canonical NRF2-pathway alterations.

We next evaluated whether mutation status modified the association between the ZIP-active score and clinical outcome. When survival analyses were performed separately according to *NFE2L2/KEAP1/CUL3* mutation status, no significant differences in overall or progression-free survival were observed between ZIP-active-high and ZIP-active-low tumors in the wild-type subgroup (Figure 4C). Similarly, no significant survival differences were detected in the mutant subgroup (Supplementary Figure S3C). In addition, *NFE2L2/KEAP1/CUL3* mutation status itself was not significantly associated with overall or progression-free survival (Supplementary Figure S3B). These findings suggest that the adverse prognostic association of the ZIP-active program observed in the overall cohort was not independently reproduced after stratification by NRF2-pathway mutation status.

To determine whether the biological characteristics associated with the ZIP-active program were preserved according to *NFE2L2*/*KEAP1/CUL3* mutation status, GSEAs were performed separately in wild-type and mutant tumors. In the wild-type subgroup, ZIP-active-high tumors were enriched for oxidative stress- and metabolism-related pathways, including reactive oxygen species and oxidative phosphorylation programs, whereas several immune- and inflammation-related pathways were preferentially enriched in ZIP-active-low tumors (Figure 4D). The mutant subgroup also showed enrichment of multiple metabolic and oxidative stress-related pathways in ZIP-active-high tumors, although the specific enrichment patterns differed between the two mutation groups (Supplementary Figure S3D). Thus, the association between ZIP activity and metabolic/oxidative stress programs was observed in both wild-type and mutant tumors rather than being confined to either genomic background.

Finally, we investigated whether the association between the ZIP-active program and the tumor immune microenvironment differed according to mutation status. T-cell-related immune signatures were reduced in ZIP-active-high tumors within the wild-type subgroup, whereas these differences were less evident in mutant tumors (Figure 4E,F and Supplementary Figure S3E,F). CIBERSORTx analysis likewise demonstrated mutation-status-dependent differences in the immune-cell composition associated with ZIP activity (Figure 4G and Supplementary Figure S3G). Together, these findings indicate that canonical NRF2-pathway alterations are associated with higher basal ZIP-active and NRF2-related activity, whereas the relationship between ZIP activity and NRF2-related metabolic programs persists in both wild-type and mutant tumors. In contrast, the immune and prognostic associations of the ZIP-active program appear more context-dependent.

## 4. Discussion

In the present study, we identified a previously unrecognized ZIP-active epithelial tumor cell state in HNSCC characterized predominantly by coordinated upregulation of *SLC39A3* and *SLC39A4*. Integrative analyses of single-cell and bulk transcriptomic datasets consistently demonstrated that this state was associated with NRF2-related oxidative stress adaptation, enhanced glutathione metabolism, transcriptional programs compatible with ferroptosis suppression, reduced T-cell-related immune signatures, and poorer overall survival. Importantly, *NFE2L2/KEAP1/CUL3*-mutant tumors exhibited higher ZIP-active and NRF2-related activity, while associations between the ZIP-active program and NRF2-related metabolic features were observed in both wild-type and mutant tumors, suggesting that the ZIP-active program represents a biologically distinct transcriptional state beyond canonical genetic activation of the NRF2 pathway.

Previous studies have implicated individual ZIP transporters, particularly *SLC39A4*, in tumor progression across several malignancies [10,12,13]. Our findings extend these observations by demonstrating that the biological significance of zinc transport in HNSCC is more accurately captured by a coordinated transcriptional program than by expression of individual transporter genes. The convergence of differential expression, pathway enrichment, transcription factor inference, pathway scoring, and trajectory analyses consistently linked the ZIP-active state to NRF2 activation, indicating that coordinated ZIP transporter expression identifies a tumor cell population adapted to oxidative stress. Interestingly, the ZIP-active phenotype was inversely associated with EMT-related transcriptional features despite its association with adverse prognosis. This finding suggests that the aggressive biological features of ZIP-active tumors may reflect NRF2-associated metabolic and oxidative stress adaptation rather than a conventional EMT-dominant phenotype. Because this study is based on transcriptomic analyses, the present findings should be interpreted as evidence of a biological association rather than a direct mechanistic ZIP–NRF2 signaling axis.

One of the most notable findings of this study is the close association between the ZIP-active state and ferroptosis-related transcriptional programs. Although ferroptosis is fundamentally an iron-dependent form of regulated cell death, ZIP-active tumor cells consistently exhibited enrichment of glutathione metabolism and ferroptosis-suppressive signatures across both single-cell and bulk transcriptomic analyses [26,27]. Since NRF2 is a major regulator of antioxidant defense and glutathione metabolism, these findings support the concept that ZIP-active tumor cells acquire a transcriptional landscape compatible with enhanced resistance to oxidative and lipid peroxidation [15,21]. Beyond glutathione metabolism, NRF2-associated antioxidant defense involves broader ROS-detoxifying systems, including superoxide dismutases, which may further contribute to oxidative stress adaptation [15,17,28]. Particularly intriguing is that this phenotype emerged from increased expression of zinc transporters rather than genes directly involved in iron metabolism, raising the possibility that altered zinc homeostasis contributes to the establishment of a ferroptosis-resistant tumor cell state. However, our data do not demonstrate a direct functional interaction between zinc transport and ferroptosis, and experimental validation using intracellular metal measurements and ferroptosis assays will be necessary to clarify this relationship.

Another important observation is that the ZIP-active program was consistently associated with an immune-cold phenotype. ZIP-active-high tumors exhibited reduced T-cell-related immune signatures together with diminished inflammatory pathway activity, and CIBERSORTx analysis further demonstrated differences in immune-cell composition, including reduced CD8 T-cell and CD4 memory T-cell fractions, supporting a close association between oxidative stress adaptation and immune suppression in ZIP-active-high tumors [29,30,31]. Emerging evidence has linked persistent NRF2 activation with impaired antitumor immunity, suggesting that metabolic adaptation may influence not only tumor cell survival but also the surrounding immune microenvironment [23,32]. Our findings therefore extend these observations by identifying a transcriptional program that simultaneously integrates redox adaptation, ferroptosis-related defense, and immune alterations in HNSCC.

The analyses according to *NFE2L2*/*KEAP1/CUL3* mutation status provide additional insight into the biological context of the ZIP-active program. Consistent with previous studies, tumors harboring canonical NRF2-pathway mutations exhibited higher baseline ZIP-active and NRF2-related signature scores [17,20,24]. Importantly, NRF2-related metabolic features, including oxidative stress- and metabolism-associated transcriptional programs, remained associated with the ZIP-active phenotype in both wild-type and mutant tumors. In contrast, immune-related associations showed greater variation according to mutation status, and the prognostic association of the ZIP-active score observed in the overall cohort was not significant when wild-type and mutant tumors were analyzed separately. Together, these findings suggest that canonical NRF2-pathway mutations augment the ZIP-active/NRF2-associated phenotype but do not fully account for its presence or its associated metabolic features. The ZIP-active program therefore appears to capture a broader transcriptional state that cannot be fully explained by canonical genetic activation of the NRF2 pathway alone.

From a clinical perspective, its association with poor prognosis and NRF2-related metabolic features in the TCGA cohort suggests that it may represent a clinically relevant biomarker of oxidative stress adaptation in HNSCC. Furthermore, its consistent association with glutathione metabolism and ferroptosis-suppressive programs raises the possibility that ZIP-active tumors may display distinct vulnerabilities to therapies targeting redox homeostasis or ferroptosis-defense pathways [33,34,35]. Although these hypotheses require functional validation, the present findings provide a rationale for investigating ZIP transporter-associated transcriptional programs as potential biomarkers for patient stratification and for identifying tumors with therapeutically relevant redox and metabolic vulnerabilities.

This study has several limitations. First, the analyses were based on retrospective transcriptomic datasets, and external validation in independent patient cohorts is warranted. Second, intracellular zinc concentrations, zinc transporter activity, NRF2 activation, and ferroptosis susceptibility were inferred from transcriptional data rather than directly measured. Third, the biological functions of *SLC39A3* and *SLC39A4* require experimental validation using genetic perturbation and functional assays. Fourth, the relatively small number of tumors harboring *NFE2L2*/*KEAP1/CUL3* mutations may have limited the statistical power of subgroup analyses. Finally, the datasets analyzed in this study did not include longitudinal paired transcriptomic samples obtained before and after treatment, precluding assessment of the temporal stability or treatment-associated changes in the ZIP-active transcriptional program.

## 5. Conclusions

We identified a previously unrecognized ZIP-active epithelial tumor cell state in HNSCC characterized by coordinated ZIP transporter expression and an NRF2-associated oxidative stress-adaptive transcriptional program. The ZIP-active state was associated with oxidative stress adaptation, immune alterations, and adverse prognosis, while NRF2-related metabolic features remained associated with ZIP activity across *NFE2L2/KEAP1/CUL3* mutation groups. These findings suggest that the ZIP-active state reflects NRF2-related tumor biology beyond genomic alterations alone.

## Figures and Tables

**Figure 1:**
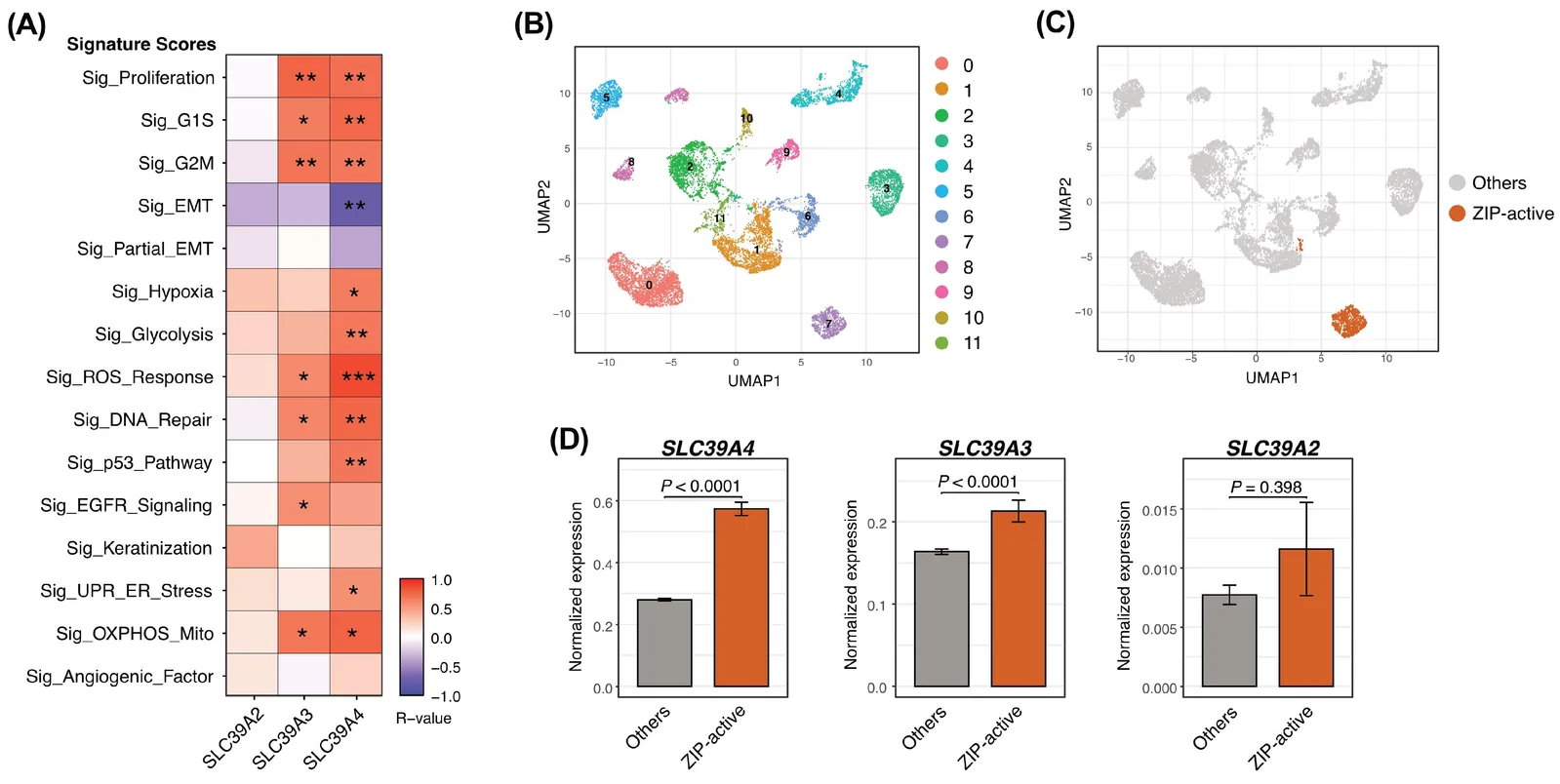
Identification of the ZIP-active epithelial tumor cell state in HNSCC by single-cell RNA sequencing. (**A**) Correlation heatmap between the expression of *SLC39A2*, *SLC39A3*, and *SLC39A4* and representative cancer-related signature scores in epithelial tumor cells from the GSE164690 cohort. (**B**) UMAP of epithelial tumor cells showing the identified epithelial subclusters. (**C**) UMAP highlighting epithelial cluster 7, designated as the ZIP-active tumor cell state. (**D**) Comparison of *SLC39A2*, *SLC39A3*, and *SLC39A4* expression between ZIP-active and other epithelial tumor cells. UMAP, Uniform Manifold Approximation and Projection; HNSCC, head and neck squamous cell carcinoma. *, *p* < 0.05; **, *p* < 0.01; ***, *p* < 0.001.

**Figure 2:**
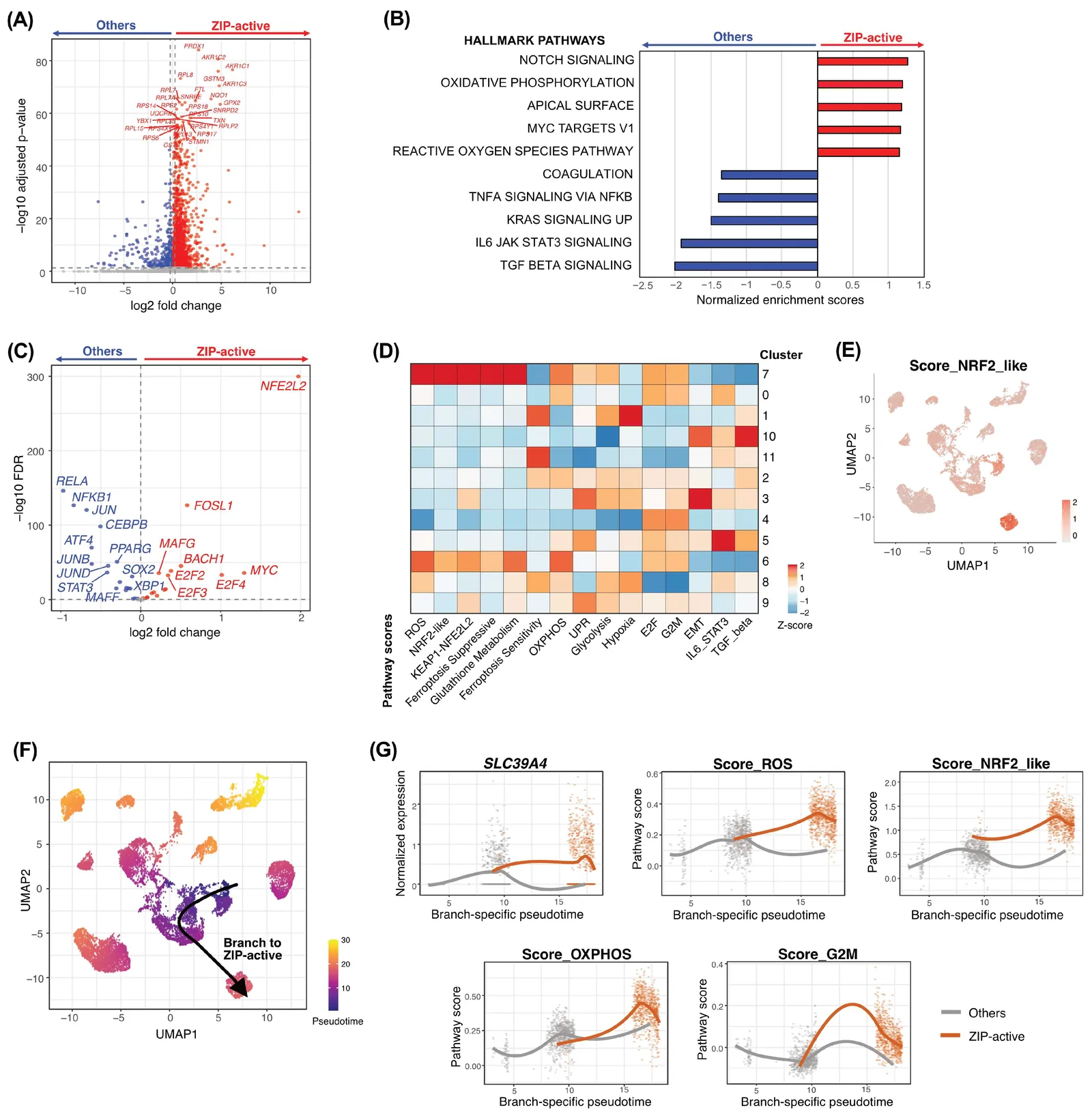
Molecular characterization of the ZIP-active epithelial tumor cell state. (**A**) Volcano plot showing differentially expressed genes between ZIP-active and other epithelial tumor cells. (**B**) GSEA of Hallmark pathways comparing ZIP-active and other epithelial tumor cells. (**C**) Volcano plot of transcription factor (TF) activity inferred by DoRothEA and VIPER analysis. (**D**) Heatmap showing pathway scores across epithelial tumor cell clusters. Pathway scores were calculated using predefined gene sets. (**E**) UMAP showing the distribution of the NRF2-like pathway score in epithelial tumor cells. (**F**) Pseudotime trajectory inferred by Slingshot, showing differentiation trajectories toward the ZIP-active tumor cell state. (**G**) Pseudotime dynamics of representative genes and pathway scores comparing ZIP-active and other epithelial tumor cells. GSEA, gene set enrichment analysis; TF, transcription factor.

**Figure 3:**
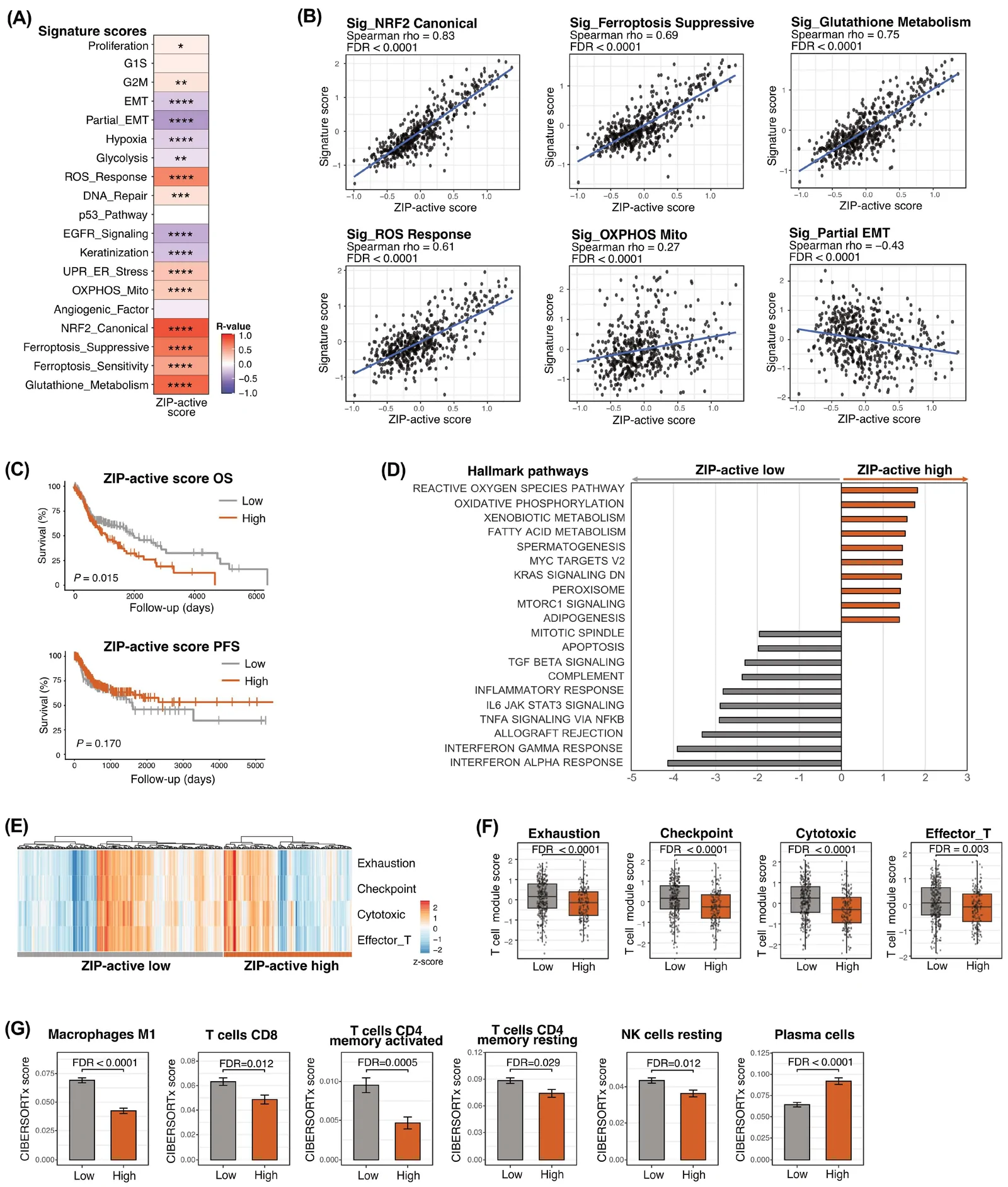
Validation of the ZIP-active transcriptional program in the TCGA-HNSCC cohort. (**A**) Correlation heatmap between the ZIP-active score and representative cancer signature scores in the TCGA-HNSCC cohort. Correlations were evaluated using Spearman’s rank correlation. (**B**) Scatter plots showing the correlations between the ZIP-active score and representative cancer signature scores. (**C**) Kaplan–Meier analyses of overall survival (OS) and progression-free survival (PFS) according to the ZIP-active score. Patients were classified into ZIP-active-high and ZIP-active-low groups using the optimal receiver operating characteristic (ROC)-based cutoff. (**D**) GSEA of Hallmark pathways comparing ZIP-active-high and ZIP-active-low tumors. (**E**,**F**) Comparison of representative T-cell-related immune signature scores between ZIP-active-high and ZIP-active-low tumors. (**G**) Comparison of immune-cell fractions estimated by CIBERSORTx between ZIP-active-high and ZIP-active-low tumors. OS, overall survival; PFS, progression-free survival; ROC, receiver operating characteristic; FDR, false discovery rate. *, FDR < 0.05; **, FDR < 0.01; ***, FDR < 0.001, ****, FDR < 0.0001.

**Figure 4:**
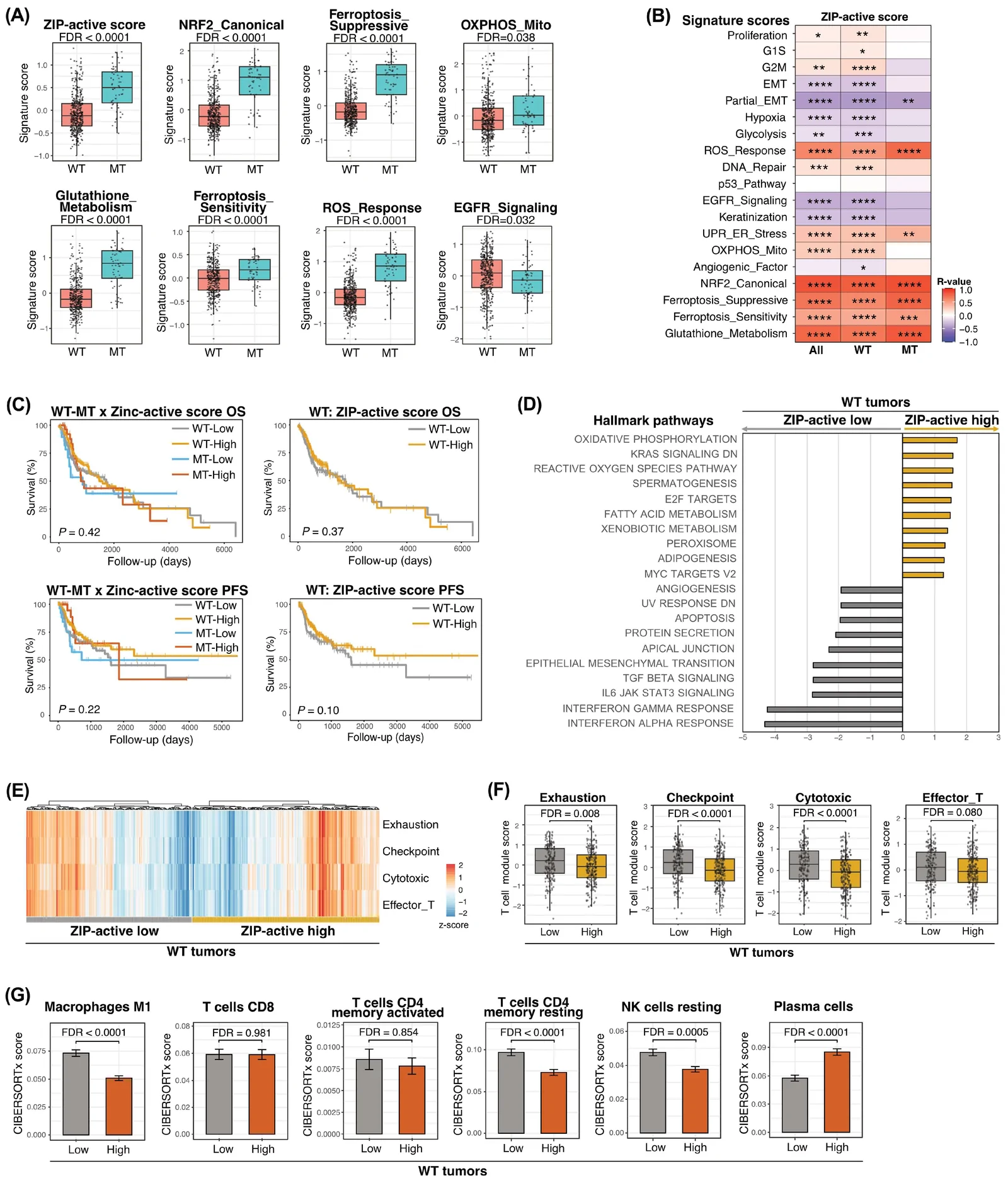
*NFE2L2*/*KEAP1/CUL3* mutation status modifies the biological and clinical features associated with the ZIP-active program. (**A**) Comparison of the ZIP-active score and representative cancer-related signature scores, including NRF2-related, glutathione metabolism, ferroptosis suppressive, ROS response, oxidative phosphorylation (OXPHOS), EGFR signaling, and p53 pathway signatures, between wild-type (WT) and mutant (MT) tumors. (**B**) Correlation heatmap showing the associations between the ZIP-active score and representative cancer-related signature scores in all tumors, WT tumors, and MT tumors. (**C**) Kaplan–Meier analyses of overall survival (OS) and progression-free survival (PFS) according to the ZIP-active score in the overall cohort and the WT subgroup. (**D**) GSEA comparing Hallmark pathways between ZIP-active-high and ZIP-active-low tumors in the WT subgroup. (**E**,**F**) Comparison of representative T-cell-related immune signature scores between ZIP-active-high and ZIP-active-low tumors in the WT subgroup. (**G**) Comparison of immune-cell fractions estimated by CIBERSORTx between ZIP-active-high and ZIP-active-low tumors within the WT subgroup. WT, wild type; MT, mutant; *, FDR < 0.05; **, FDR < 0.01; ***, FDR < 0.001, ****, FDR < 0.0001.

**Table 1:** Univariate and multivariate survival analyses of OS and PFS in patients with HNSCC.

Variables	Overall Survival			Progression-Free Survival		
	Univariate	Multivariate		Univariate	Multivariate	
	*p*-Value	HR (95% CI)	*p*-Value	*p*-Value	HR (95% CI)	*p*-Value
HPV status (ref: negative)						
Positive	0.137			0.050	0.56 (0.34–0.94)	0.027
Primary lesion (ref: oral cavity)						
Oropharynx	0.045	0.48 (0.23–0.97)	0.042	0.149		
Larynx	0.444	0.71 (0.49–1.04)	0.076	0.250		
Hypopharynx	0.395	1.22 (0.45–3.33)	0.696	0.215		
TNM stage (ref: I–II)						
III–IV	0.010	1.65 (1.12–2.43)	0.011	0.011	1.98 (1.21–3.24)	0.006
ZIP-active score (ref: low)						
High	0.015	1.40 (1.03–1.89)	0.031	0.170		

Abbreviations: PFS, progression-free survival; OS, overall survival; HNSCC, head and neck squamous cell carcinoma; HPV, human papillomavirus; HR, hazard ratio; CI, confidence interval; ref, reference.

## Data Availability

The original data presented in this study are openly available in GSE164690, https://www.ncbi.nlm.nih.gov/geo/query/acc.cgi?acc=GSE164690 (accessed on 1 June 2023), and on the FireBrowse website, http://firebrowse.org/ (accessed on 6 May 2020).

## Supplementary Materials

The following supporting information can be downloaded at: https://www.mdpi.com/article/10.3390/cancers18193104/s1, Figure S1: Additional characterization of the ZIP-active epithelial tumor cell state; Figure S2: Additional analyses of ZIP-active score associations in the TCGA-HNSCC cohort; Figure S3: Additional subgroup analyses according to *NFE2L2*/*KEAP1/CUL3* mutation status in the TCGA-HNSCC cohort; Table S1: Clinical characteristics of patients included in the single-cell RNA sequencing analysis; Table S2: Gene sets used for cancer signature scoring [4,36,37,38,39,40,41,42,43,44]; Table S3: Gene sets used for pathway score calculation shown in Figure 2D; Table S4: Gene sets used for T-cell signature scoring [45,46,47,48,49,50]; Table S5: Association between ZIP-active score and clinicopathological features in the TCGA cohort of 520 patients with HNSCC.

## Abbreviations

The following abbreviations are used in this manuscript:

ANOVA	analysis of variance
CI	confidence interval
EGFR	epidermal growth factor receptor
FDR	false discovery rate
GSEA	gene set enrichment analysis
HNSCC	head and neck squamous cell carcinoma
HPV	human papillomavirus
HR	hazard ratio
MSigDB	Molecular Signatures Database
MT	mutant
NRF2	nuclear factor erythroid 2-related factor 2
OS	overall survival
OXPHOS	oxidative phosphorylation
PFS	progression-free survival
ROC	receiver operating characteristic
ROS	reactive oxygen species
scRNA-seq	single-cell RNA sequencing
TCGA	The Cancer Genome Atlas
TF	transcription factor
TNM	tumor–node–metastasis
UMAP	Uniform Manifold Approximation and Projection
WT	wild type
ZIP	Zrt-/Irt-like protein
ZnT	zinc transporter
