# Supplementary material for: A ZIP-Active Tumor Cell State Defines an NRF2-Associated Ferroptosis-Resistant and Immune-Cold Transcriptional Program in Head and Neck Squamous Cell Carcinoma
Source: Cancers (Basel). 2026 Sep 24;18(19):3104. doi: 10.3390/cancers18193104 (PMC13663997; doi:10.3390/cancers18193104)
Supplement: Supplementary file 1 [file cancers-18-03104-s001.zip › cancers-4526745-supplementary.pdf]

cancers

MDPI

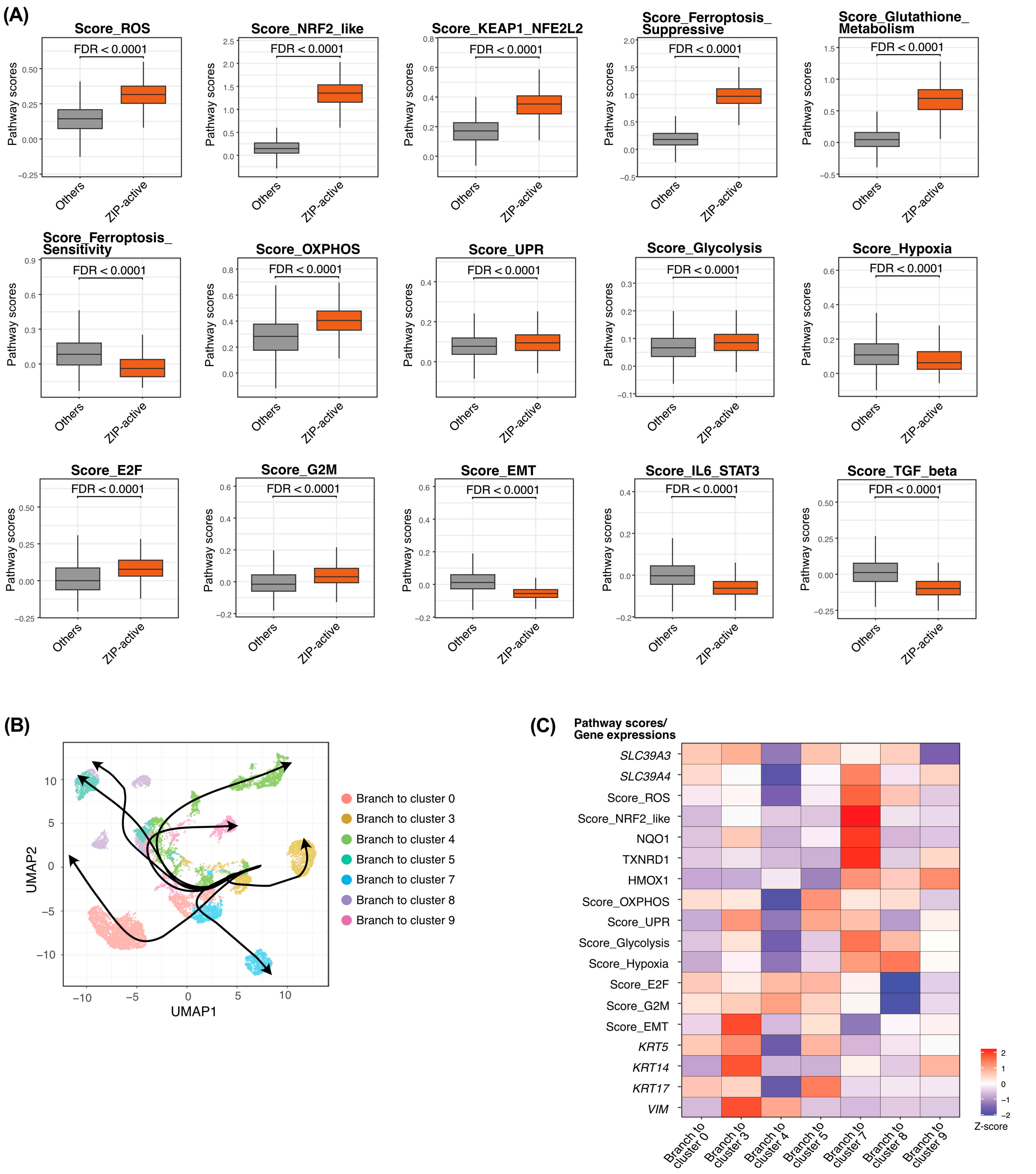

Figure S1. Additional characterization of the ZIP-active epithelial tumor cell state. (A) Comparison of pathway scores between the ZIP-active tumor cell state and other epithelial tumor cells. Bar plots show the average module scores of representative biological pathways. (B) Slingshot trajectory analysis of epithelial tumor cells. UMAP visualization showing the seven inferred differentiation branches. (C) Heatmap summarizing pathway scores and representative gene expression across the seven Slingshot branches, illustrating the molecular characteristics associated with each differentiation trajectory.

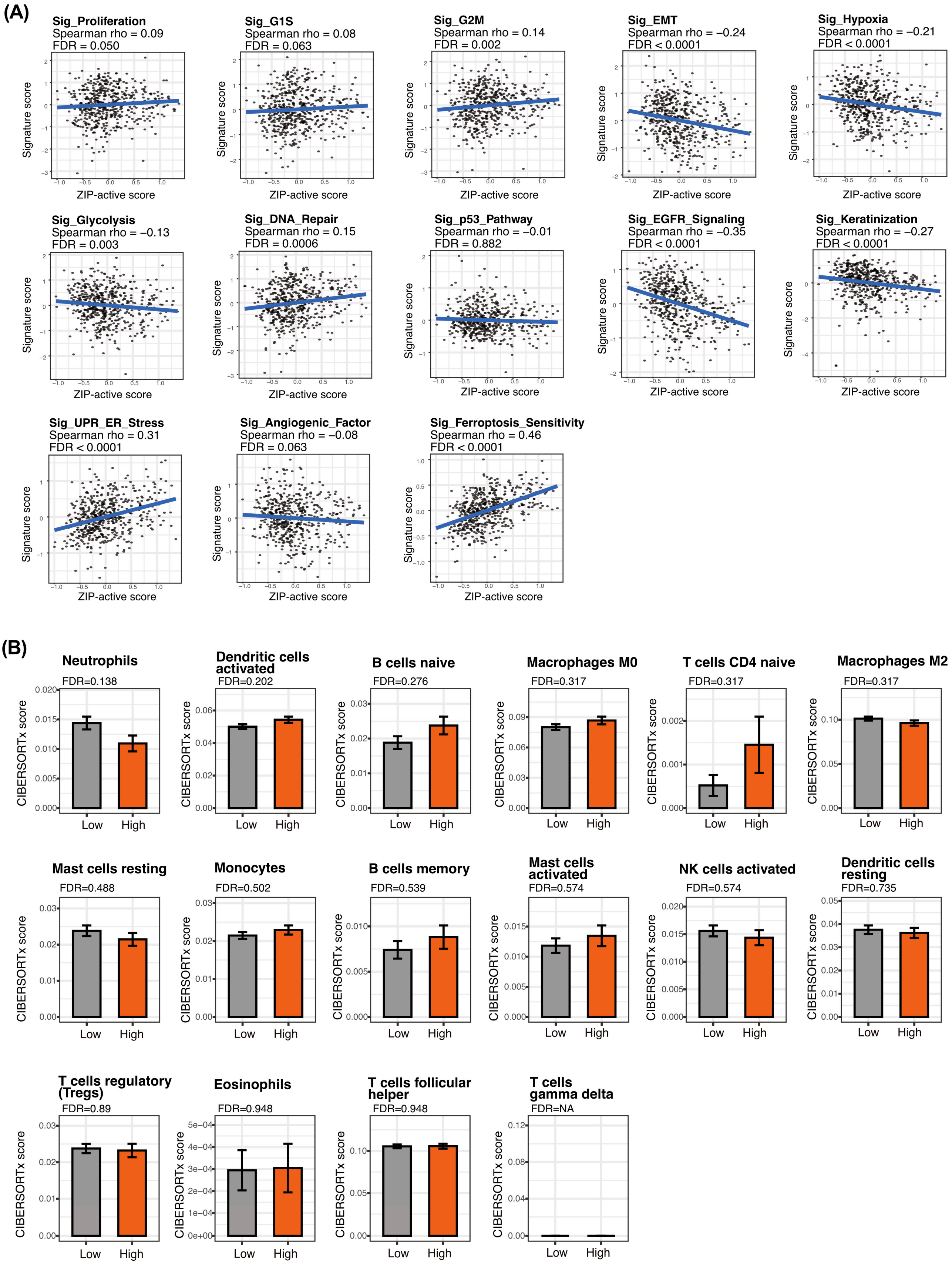

Figure S2. Additional analyses of ZIP-active score associations in the TCGA-HNSCC cohort. (A) Scatter plots showing the correlations between the ZIP-active score and representative cancer-related signature scores in the TCGA HNSCC cohort (additional data to Figure 3B). (B) Comparison of immune cell fractions estimated by CIBERSORTx between ZIP-active-high and ZIP-active-low tumors (additional data to Figure 3G).

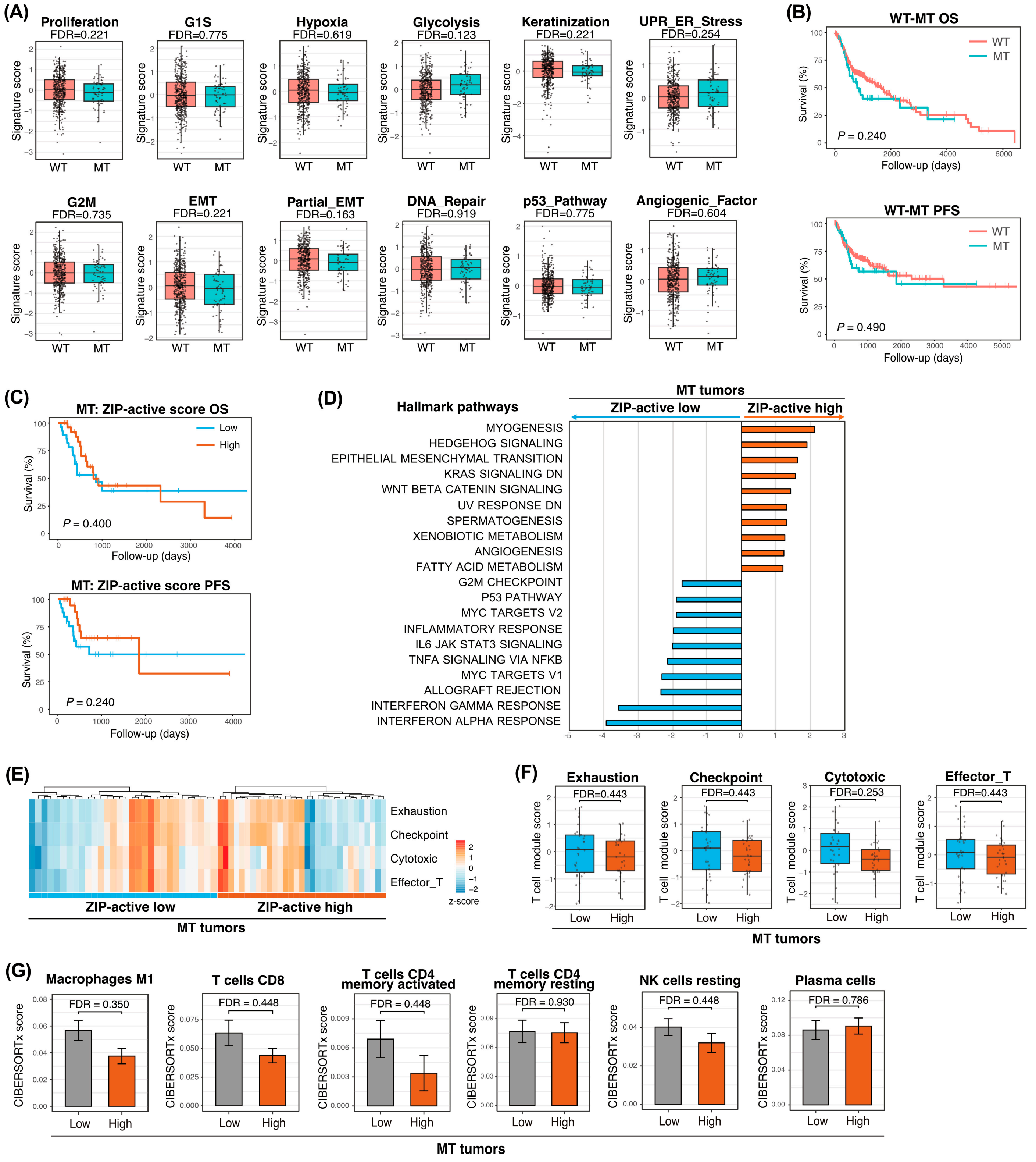

Figure S3. Additional subgroup analyses according to *NFE2L2/KEAP1/CUL3* mutation status in the TCGA-HNSCC cohort. (A) Comparison of representative cancer-related signature scores between tumors in the wild-type (WT) and mutant (MT) tumors (additional data to Figure 4A). (B) Kaplan-Meier analyses of overall survival (OS) and progression-free survival (PFS) comparing patients with WT and MT tumors. (C) Kaplan-Meier analyses of OS and PFS according to the ZIP-active score in the MT subgroup. (D) Gene set enrichment analysis (GSEA) comparing Hallmark pathways between ZIP-active-high and ZIP-active-low tumors in the MT subgroup. (E,F) Comparison of representative T-cell-related immune signature scores between ZIP-active-high and ZIP-active-low tumors in

the MT subgroup. (G) Comparison of immune cell fractions estimated by CIBERSORTx between ZIP-active-high and ZIP-active-low tumors in the MT subgroup.

Table S1. Clinical characteristics of patients included in the single-cell RNA sequencing analysis

Patient	Sex	Age group	Primary site	T factor	N factor	M factor	HPV status	Smoking	Alcohol
1	M	70-79	Oral cavity	T4A	N2B	M0	Neg	Yes	No
5	F	50-59	Oral cavity	T3	N3b	M0	Neg	Yes	Yes
6	M	30-39	Oral cavity	T3	N0	M0	Neg	Yes	Yes
7	M	60-69	Larynx	T3	N0	M0	Neg	Yes	Yes
8	F	70-79	Oral cavity	T1	N0	M0	Neg	Yes	Yes
9	F	70-79	Oral cavity	T3	N2B	M0	Neg	Yes	Yes
11	M	80-89	Oral cavity	T2	N0	M0	Neg	No	No
12	M	50-59	Oropharynx	T2	N1	M0	Pos	Yes	Yes
13	M	70-79	Oropharynx	T2	N0	M0	Pos	No	No
14	M	50-59	Oropharynx	T1	N1	M0	Pos	Yes	Yes
15	F	60-69	Oral cavity	T2	N0	M0	Neg	Yes	NA
16	M	40-49	Oropharynx	T2	N1	M0	Pos	Yes	Yes
17	M	50-59	Oropharynx	T1	N1	M0	Pos	Yes	Yes
18	M	50-59	Oropharynx	T2	N2	M0	Pos	Yes	Yes

Clinical information was obtained from the original report by Kürten et al. Single-cell RNA sequencing was performed using cells isolated from freshly resected HNSCC specimens.

Table S2. Gene sets used for cancer signature scoring.

**Signature**	**Genes**	**Reference**
Proliferation	*MKI67, TOP2A, PCNA, MCM2, MCM4, MCM6, UBE2C, BIRC5*	Whitfield ML *et al.* Nat Rev Cancer, 2006 [36]
G1/S cell cycle	*MCM5, PCNA, TYMS, FEN1, MCM2, MCM4, RRM1, RRM2*	Whitfield *et al.* Nat Rev Cancer, 2006 [36]
G2/M cell cycle	*HMGB2, CDK1, NUSAP1, UBE2C, BIRC5, TOP2A, NDC80, CKS2*	Whitfield *et al.* Nat Rev Cancer, 2006 [36]
EMT	*VIM, FN1, SNAI1, SNAI2, ZEB1, ZEB2, TWIST1, ITGA5, ITGB1*	Thiery JP *et al.* Cell, 2009 [37]
Partial EMT	*LAMC2, LAMB3, ITGA3, ITGB4, VIM, TGFBI, PDPN, MMP10*	Puram SV *et al.* Cell, 2017 [4]
Hypoxia	*HIF1A, VEGFA, CA9, SLC2A1, LDHA, PGK1, ENO1, BNIP3*	Liberzon *et al.* Cell Systems, 2015 (Hallmark Hypoxia) [38]
Glycolysis	*SLC2A1, HK2, PFKP, ALDOA, GAPDH, PGK1, ENO1, PKM, LDHA*	Liberzon *et al.* Cell Systems, 2015 (Hallmark Glycolysis) [38]

ROS response	*HMOX1, NQO1, TXNRD1, GCLC, GCLM, SOD1, SOD2, PRDX1*	Liberzon *et al.* Cell Systems, 2015 (Hallmark Reactive Oxygen Species Pathway) [38]
DNA repair	*BRCA1, BRCA2, RAD51, CHEK1, PARP1, MCM2, MCM6, RPA1*	Liberzon *et al.* Cell Systems, 2015 (Hallmark DNA Repair) [38]
p53 pathway	*CDKN1A, MDM2, BBC3, BAX, GADD45A, DDB2, RRM2B*	Liberzon *et al.* Cell Systems, 2015 (Hallmark p53 Pathway) [38]
EGFR signaling	*EGFR, EREG, AREG, HBEGF, DUSP6, FOS, JUN, MYC*	Yarden Y & Sliwkowski MX Nat Rev Mol Cell Biol, 2001 [39]
Keratinization	*KRT5, KRT6A, KRT6B, KRT14, KRT16, KRT17, IVL, SPRR1B, SPRR2A, DSG3*	Puram *et al.* Cell, 2017 [4]
UPR / ER stress	*HSPA5, ATF4, ATF6, XBP1, DDIT3, ERN1, EIF2AK3, DNAJB9, HERPUD1*	Liberzon *et al.* Cell Systems, 2015 (Hallmark Unfolded Protein Response) [38]
OXPHOS / Mitochondria	*NDUFA1, NDUFB8, SDHB, UQCRC1, COX5A, COX6C, ATP5F1A, ATP5MC1*	Liberzon *et al.* Cell Systems, 2015 (Hallmark Oxidative Phosphorylation) [38]
Angiogenic factor	*VEGFA, ANGPTL4, PGF, PDGFA, PDGFB, JAG1, CXCL8, IL6*	Liberzon *et al.* Cell Systems, 2015 (Hallmark Angiogenesis) [38]
NRF2 canonical	*NQO1, TXNRD1, HMOX1, GCLC, GCLM, GPX2, PRDX1, AKR1C1, AKR1C2, AKR1C3, GSTA1, GSTA4, GSTM1, GSTM3, UGT1A6, UGT1A9, OSGIN1*	Kensler et al., Annu Rev Pharmacol Toxicol, 2007; Hayes and Dinkova-Kostova, Trends Biochem Sci, 2014 [40,41]
Ferroptosis suppressive	*SLC7A11, SLC3A2, GPX4, FSP1/AIFM2, GCLC, GCLM, GSS, GSR, FTH1, FTL, NQO1, HMOX1, TXNRD1, SCD, PROM2, AKR1C1, AKR1C2, AKR1C3*	Stockwell et al., Cell, 2017; Bersuker et al., Nature, 2019 [42,43]
Ferroptosis sensitivity	*ACSL4, LPCAT3, ALOX5, ALOX12, ALOX15, TFRC, SAT1, CHAC1, PTGS2, NOX1, NOX4, DPP4, BID, BECN1*	Stockwell et al., Cell, 2017; Jiang et al., Nat Rev Mol Cell Biol, 2021 [42,44]
Glutathione metabolism	*SLC7A11, SLC3A2, GCLC, GCLM, GSS, GSR, GPX4, GPX2, GSTP1, GSTA1, GSTA4, TXNRD1, NQO1*	Stockwell et al., Cell, 2017; Jiang et al., Nat Rev Mol Cell Biol, 2021 [42,44]
ZIP-active score	*A1BG, ABCC1, ACTL8, ADAM23, ADH7, AKR1B10, AKR1C1, AKR1C2, AKR1C3, ALDH3A1, MEIOB, COA6, SAPCD2, CBR1, CCDC58, CENPV, CES1, COCH, DCUN1D1, DLK2, DMKN, DMRT2, EID3, EIF5A2, EPHX1, F12, C1QTNF12, FBXO27, FTL, G6PD, GAMT, GCSH, GFRA3, GPC3, GPX2, GSTA1, GSTA4, GSTM1, GSTM3, GSTM4, GSTO2, H2AFY2, ICK, IL17RE, ITPR1, KIAA1324, LGALS7B, MAP1B, MATN2, MCCC1, ME1, MESP1, C8orf82, MYH11, NAA38, NMB, NPDC1, NPW, NQO1, NTS, OSGIN1, PANX2, PARL, PCSK1N, PGD, POMC, PRDX1, PRSS21, RAB3B, RAB6B, RAC3, RBP7, REC8, RGS9, SOX15, SOX2, SPP1, THOC3, TMEM116, TMEM14A, TNFRSF18, TNIK, TNNI3, TPRG1, TRIM16L, TSPAN7, TXNRD1, UCHL1*	The present study

Table S3. Gene sets used for pathway score calculation shown in Figure 2D.

Pathway score	Gene set source	Genes
Score_ROS	MSigDB Hallmark (HALLMARK_REACTIVE_OXYGEN_SPECIES_PATHWAY)	Entire Hallmark ROS pathway gene set detected in the dataset
Score_OXPHOS	MSigDB Hallmark (HALLMARK_OXIDATIVE_PHOSPHORYLATION)	Entire Hallmark OXPHOS gene set detected in the dataset
Score_UPR	MSigDB Hallmark (HALLMARK_UNFOLDED_PROTEIN_RESPONSE)	Entire Hallmark UPR gene set detected in the dataset *
Score_Glycolysis	MSigDB Hallmark (HALLMARK_GLYCOLYSIS)	Entire Hallmark Glycolysis gene set detected in the dataset *
Score_Hypoxia	MSigDB Hallmark (HALLMARK_HYPOXIA)	Entire Hallmark Hypoxia gene set detected in the dataset *
Score_E2F	MSigDB Hallmark (HALLMARK_E2F_TARGETS)	Entire Hallmark E2F Targets gene set detected in the dataset *
Score_G2M	MSigDB Hallmark (HALLMARK_G2M_CHECKPOINT)	Entire Hallmark G2M Checkpoint gene set detected in the dataset *
Score_EMT	MSigDB Hallmark (HALLMARK_EPITHELIAL_MESENCHYMAL_TRANSITION)	Entire Hallmark EMT gene set detected in the dataset *
Score_MYC	MSigDB Hallmark (HALLMARK_MYC_TARGETS_V1)	Entire Hallmark MYC Targets V1 gene set detected in the dataset *
Score_p53	MSigDB Hallmark (HALLMARK_P53_PATHWAY)	Entire Hallmark p53 pathway gene set detected in the dataset *
Score_Apoptosis	MSigDB Hallmark (HALLMARK_APOPTOSIS)	Entire Hallmark Apoptosis gene set detected in the dataset *
Score_IL6_STAT3	MSigDB Hallmark (HALLMARK_IL6_JAK_STAT3_SIGNALING)	Entire Hallmark IL6/JAK/STAT3 signaling gene set detected in the dataset *
Score_TGF_beta	MSigDB Hallmark (HALLMARK_TGF_BETA_SIGNALING)	Entire Hallmark TGF-β signaling gene set detected in the dataset *
Score_Translation_Initiation	Reactome	REACTOME_EUKARYOTIC_TRANSLATION_INITIATION *
Score_Translation_Elongation	Reactome	REACTOME_EUKARYOTIC_TRANSLATION_ELONGATION *
Score_Ribosome_QC	Reactome	REACTOME_RIBOSOME_ASSOCIATED_QUALITY_CONTROL *
Score_Starvation_Response	Reactome	REACTOME_CELLULAR_RESPONSE_TO_STARVATION *
Score_EIF2AK4_AminoAcid_Deficiency	Reactome	REACTOME_RESPONSE_OF_EIF2AK4_GCN2_TO_AMINO_ACID_DEFICIENCY *
Score_NMD	Reactome	REACTOME_NONSENSE_MEDIATED_DECAY *
Score_KEAP1_NFE2L2	Reactome	REACTOME_KEAP1_NFE2L2_PATHWAY*
Score_NRF2_like	Custom	*NQO1, HMOX1, TXNRD1, GCLC, GCLM, SLC7A11, PRDX1, PRDX6, SRXN1, FTH1, FTL, AKR1C1, AKR1C2, AKR1C3, GSTA1, GSTA4, GSTM1, GSTM3, GPX2*
Score_Ferroptosis_Suppressive	Custom	*SLC7A11, SLC3A2, GPX4, FSP1 (AIFM2), GCLC, GCLM, GSS, GSR, FTH1, FTL, NQO1, HMOX1, TXNRD1, SCD, PROM2, AKR1C1, AKR1C2, AKR1C3*

Score_Ferroptosis_Sensitivity	Custom	*ACSL4, LPCAT3, ALOX5, ALOX12, ALOX15, TFRC, SAT1, CHAC1, PTGS2, NOX1, NOX4, DPP4, BID, BECN1*
Score_Glutathione_Metabolism	Custom	*SLC7A11, SLC3A2, GCLC, GCLM, GSS, GSR, GPX4, GPX2, GSTP1, GSTA1, GSTA4, TXNRD1, NQO1*
Score_ZincTransporter_Core	Custom	*SLC39A4, SLC39A3, SLC30A5, SLC30A6, SLC30A9*

* For Hallmark and Reactome signatures, module scores were calculated using all genes detected in the expression matrix from the corresponding MSigDB gene set. Custom signatures were manually curated based on the literature and zinc/ferroptosis biology.

Table S4. Gene sets used for T-cell signature scoring.

Signature	Genes	Reference
Exhaustion	*PDCD1, HAVCR2, LAG3, CTLA4, TIGIT, TOX, EOMES*	Wherry et al., Nature Reviews Immunology (2015); Scott et al., Nature (2019) [45,46]
Checkpoint	*PDCD1, HAVCR2, LAG3, CTLA4, TIGIT, BTLA, CD274, PDCD1LG2*	Jiang et al., Nature Medicine (2018) [47]
Cytotoxic	*IFNG, PRF1, GZMB, GZMA, GNLY, NKG7, CXCL9, CXCL10*	Rooney et al., Cell (2015) [48]
Effector T	*TBX21, CX3CR1, FGFBP2, PRF1, GZMB, IFNG, NKG7*	Guo et al., Nature Medicine (2018); Sade-Feldman et al., Cell (2018) [49,50]

Table S5. Association between ZIP-active score and clinicopathological features in the TCGA cohort of 520 patients with HNSCC.

**Variables**	**ZIP-active score**	
	**Mean (sem)**	***P*-value**
HPV status		
Negative	0.007 (0.023)	0.350
Positive	−0.032 (0.035)	
Primary lesion		
Hypopharynx	−0.013 (0.105)	<0.0001
Larynx	0.270 (0.043)	
Oral cavity	−0.090 (0.023)	
Oropharynx	0.012 (0.044)	
T factor		
T1-2	−0.026 (0.028)	0.267
T3-4	0.017 (0.027)	
N factor		
Negative	−0.016 (0.030)	0.427
Positive	0.016 (0.028)	
M factor		
M0	−0.006 (0.020)	0.195
M1	0.414 (0.270)	
TNM stage		
I-II	−0.076 (0.039)	0.031
III-IV	0.021 (0.022)	

Abbreviations: HNSCC, head and neck squamous cell carcinoma; HPV, human papillomavirus.
